# Electroacupuncture of 2 Hz Has a Rewarding Effect: Evidence from a Conditioned Place Preference Study in Rats

**DOI:** 10.1093/ecam/nen043

**Published:** 2011-08-09

**Authors:** Wei Xia, Ning-Ning Chu, Jing Liang, Yi-Jing Li, Rong Zhang, Ji-Sheng Han, Cai-Lian Cui

**Affiliations:** Department of Neurobiology, Neuroscience Research Institute, School of Basic Medical Sciences, Peking University, Key Lab for Neuroscience of the Ministry of Education and Key Lab for Neuroscience, Ministry of Public Health, 38 Xueyuan Road, Beijing 100191, China

## Abstract

Electroacupuncture (EA) has been used to suppress heroin craving in addicts and the conditioned place preference (CPP) for morphine in the rat. The question remained whether EA by itself will produce some rewarding effect. This was investigated using the CPP procedure in the present study. The results indicated that rats showed a significant preference to the 2 Hz EA-paired compartment. This rewarding effect of EA was prevented by pre-treatment with the opioid receptor antagonist naloxone [2 mg kg^−1^, intraperitoneally (i.p.)], CB1 cannabinoid antagonist AM251 (3 **μ**g per rat, intracerebroventricularly) or D1 dopamine receptor antagonist SCH23390 (0.1 mg kg^−1^, i.p.), respectively. TempspacetempspaceIt is concluded that 2 Hz EA is capable of inducing CPP in the rat via the activation of the endogenous opioid-, cannabinoid- and dopamine-systems.

## 1. Introduction

Electroacupuncture (EA), being more effective than the manual acupuncture [1], has been documented to be a safe, gentle and effective approach for treating various kinds of diseases or symptoms such as acute and chronic pain [2]. Our recent studies have demonstrated that EA could also serve as a potential treatment for opioid addiction. For example, EA or acupoint nerve stimulation could ameliorate withdrawal syndrome and craving for drug in heroin addicts [3–5], inhibit the expression of morphine-induced conditioned place preference (CPP) in rats [4–6] and postpone the relapse of drug reuse in detoxified former heroin addicts [5]. Notably, the EA's inhibitory effect on the expression of morphine-induced CPP could be blocked by opioid receptor antagonist naloxone (1 mg kg^−1^, intraperitoneally (i.p.)), suggesting that this effect is mediated by endogenous opioid system possibly via *μ*-receptor [7]. In addition, direct evidence has been obtained that low frequency (2 Hz) EA could increase the release of enkephalins in CNS, which interacts with *μ*- and *δ*-opioid receptors [8, 9]. Since endogenous opioid peptides in CNS are known to have rewarding effect [10, 11], we wonder if EA *per se* could induce CPP. Considering the complex network system in the CNS, the effect of EA may be mediated not only by the endogenous opioid system but also by some other related systems in the brain. Thus, the involvement of endocannabinoid system is suggested since morphine-induced CPP in rats could be blocked by SR141716A, a canabinoid receptor 1 (CB1) antagonist [12, 13], and the possible involvement of dopaminergic transmission is suggested by the fact that EA-induced CPP was blocked by D1 dopamine receptor antagonist [14]. Both the endocannabinoid system and mesolimbic dopamine system then were considered in the present study.

## 2. Methods

### 2.1. Animals

Male Sprague–Dawley rats (8- to 10-weeks-old) were obtained from the Peking University Experimental Animal Center, weighing 180–220 g at the beginning of the experiment. Animals were housed four per cage in a 12:12 h light/dark cycle (lights on at 07:00) with food and water available at all times. The room temperature was maintained at 21–23°C and relative humidity at 45–50%. Animals were conditioned and tested during the light phase of the cycle. They were handled daily during the first week after arrival. All experimental procedures were approved by the Animal Use Committee of the Peking University Health Science Center.

### 2.2. Intracerebroventricular Injection

The animals were anesthetized with sodium pentobarbital (40 mg kg^−1^, i.p.) and positioned in a Kopf stereotaxic instrument. The 24-gauge stainless steel guide cannulae were placed bilaterally to the lateral ventricles according to the atlas of Paxinos and Watson [15]. The coordinates were set as follows: 0.8 mm posterior to bregma, 1.6 mm lateral to midline and 4 mm ventral to the surface of the cortex. Cannulae were secured to the skull by jewelers' screws with dental acrylic. To prevent clogging, stainless steel stylets (27 gauge) were placed in the guide cannulae until the animals were given the intracerebroventricular (i.c.v.) injection. All animals were allowed 5 days for recovery from surgery. For drug infusion, the animals were gently restrained by hand; the stylets were removed from the guide cannulae and replaced by 27-gauge injection needles, extending the injection needle 0.5 mm below the tip of the guide cannula in the i.c.v. injection. Each i.c.v. injection unit was connected by polyethylene tubing to a 10 *μ*l Hamilton syringe. The lateral ventricles were infused with a 4 *μ*l solution (4 *μ*l/rat) over a 2-min period. The injection needle was left in place for an additional 60 s to allow diffusion, and then the stylet was reinserted into the guide cannula.

### 2.3. Drugs

Naloxone HCl, SCH23390 and eticlopride (all obtained from Sigma, USA) were dissolved in 0.9% saline, respectively and injected i.p. to the rats. AM251 (Tocris, Avonmouth Bristol, UK) was dissolved in 99.9% DMSO (Sigma, St. Louis, MO, USA) for i.c.v. injection. The injection volume was 4 *μ*l followed by a 5 *μ*l saline flush, to be completed in 3 min.

### 2.4. Conditioned Place Preference

Place conditioning was conducted in a three-compartment apparatus with an unbiased design. The apparatus was a rectangular black PVC box (75 × 22 × 30 cm^3^) divided into three chambers separated by guillotine doors. The two end chambers (30 × 22 × 30 cm^3^) used for conditioning were connected by a smaller center chamber (15 × 22 × 30 cm^3^). The two end chambers were distinguished from each other in two ways. One had a group of four lights arranged in a square pattern on the end wall and a stainless steel mesh floor (1.3 × 1.3 cm^3^), whereas the other had the lights arranged in a triangle form on the wall and a rod floor (1.3 cm apart) [16]. The center chamber had gray walls and a smooth floor. Fifteen infrared beams spaced 5 cm apart were monitoring the motion of the rat. The infrared sensors communicated to a computer every 100 ms through an interface. All experimental events were controlled and recorded automatically by the computer and the interface was located in the same room. The computer also provided continuous white noise served to mask external sounds.

The CPP procedure consisted of three phases including pre-test, conditioning and test. Prior to the start of experiment, the subjects were handled (2 min per rat) twice daily (at 8:00 A.M. and 2:00 P.M.) for 5 days. On the pre-test day (Day 0), rats were placed individually in the center chamber with the guillotine doors removed. They were allowed to freely explore the entire apparatus for a 15-min session. The amount of time spent in each compartment was recorded automatically. Rats that spent more time (over 100 s) in one of the end chambers than the other, and those that spent more than 300 s in the middle chamber were excluded from the experiment. Over the next 10 sessions (two sessions per day) subjects received a double-alternating sequence of differential conditioning. In the morning, rats were directly placed in the compartment assigned as “non-EA” for 45 min. In the afternoon (6 h later), rats were treated with EA and placed in the compartment assigned as “EA”. After each conditioning session, the rats were returned to their home cages and the entire apparatus was cleaned with alcohol wipes to minimize trapped odors. On the test day (Day 6) 24 h after the last training, rats in every group were tested under the conditions used for pre-test without any treatment [16]. Then by using a factor of correction (factor of correction = 900/(time spent in EA-paired + time spent in non-EA-paired compartment), the time spent in the central compartment was proportionally divided between the two conditioning compartments. That is to say, the corrected time in each conditioning compartment amounts to the recorded time multiplied by the factor of correction. So the total corrected time spent in both conditioning compartment always amounts to 900 s, and it is sufficient to analyse the time spent in just one compartment [17].

### 2.5. Electroacupuncture

Rats were kept in special holders with their hind legs and tails exposed [9]. Two stainless steel needles of 0.3 mm diameter were inserted into each hind leg in the acupoints ST36 (5 mm lateral to the anterior tubercle of the tibia) and SP6 (3 mm proximal to the superior border of the medial malleolus, at the posterior border of the tibia). Constant current square-wave electric stimulation was produced by a HANS LH-800 programmed pulse generator (Beijing Astronautics and Aeronautics Aviation University, Beijing, China). The frequency of EA was set at 2 Hz. The intensity was increased stepwise from 0.5 to 1 mA and ended at 1.5 mA, with each step lasting for 10 min. To control the unavoidable effects of restraint stress from EA treatment, the subjects of the restraint group were simply restrained in the holder for 30 min.

### 2.6. Data Analysis

In the CPP test, data of the time spent in EA-paired compartment were presented as mean ± SEM. One-way analysis of variance (ANOVA) was used to analyze the time spent in the target compartment. When significant differences were found, *post hoc* analyses were conducted using Student-Newman-Keul's test. The accepted level of statistical significance is *P* <  .05.

## 3. Results

The pre-conditioning test showed that most animals (75%) spent an equal amount of time in the two end chambers and less time in the small center choice chamber. After the pre-test, a few rats (25%) that spent more time (over 100 s) in one of the end chambers than the other and that spent >300 s in the middle chamber were excluded from the experiment, so the rats used in our research were truly unbiased in terms of chamber preferences (data not shown).

### 3.1. EA *per se* could Induce CPP

**Figure 1:**
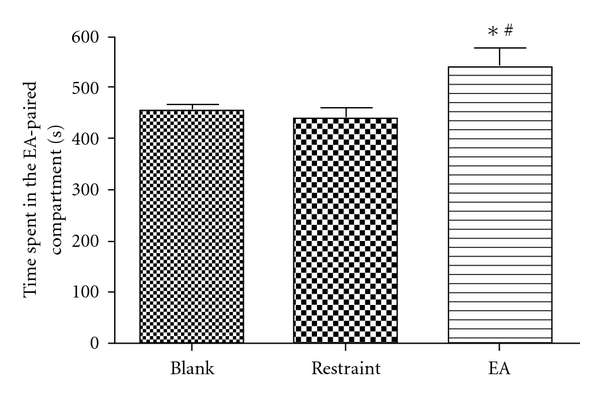
CPP induced by EA. *P <  .05, compared with the blank group; ^#^ *P* <  .05, compared with the restraint group (*n* = 12–15). One-way ANOVA followed by Student-Newman-Keul's test.

On the unbiased condition, rats were divided into three groups: blank group, restraint group and EA group. Rats of blank group received no treatment prior to each conditioning session, rats in restraint group were restrained in holders for 30 min without EA and rats in EA group were treated with EA for 30 min prior to conditioning as described in Section 2. As shown in Figure 1, the rats received EA treatment spent significantly more time in EA-paired compartment than those of blank and restraint group (*P* <  .05, one-way ANOVA followed by Student-Newman-Keul's test) after 5 days of conditioning.

### 3.2. Endogenous Opioid Implicated in EA-Induced CPP

**Figure 2:**
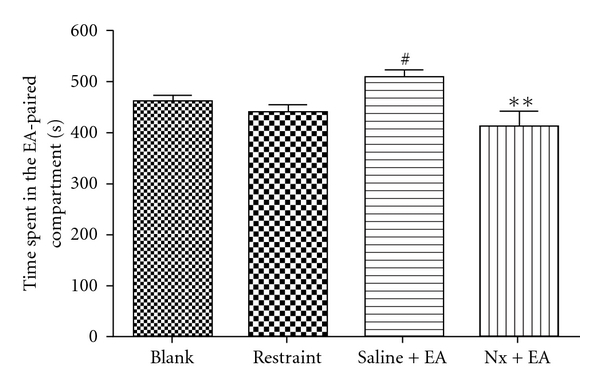
EA-induced CPP in rats was suppressed by naloxone (2 mg/kg). **P <  .01, compared with saline + EA group; ^#^ *P* <  .05, compared with the restraint group; (*n* = 10–15). One-way ANOVA followed by Student-Newman-Keul's test.

To clarify if endogenous opioid implicated in EA-induced CPP development, we test first if naloxone administration, 45 min before placing into the CPP training chamber, can induce aversive properties. Twenty-four rats were randomly divided two groups (naloxone and normal saline). After the conditioning for 5 days, results showed that rats in naloxone treatment could not display significant CPA properties compared with the normal saline group (data not shown). Second, another two groups of rats were conditioned with EA for 5 days. One group (Nx + EA) received naloxone (2 mg/kg) 15 min before each EA treatment, the other received the saline injection (Saline + EA). blank group and restraint group were used as controls.  Figure 2 shows that the establishment of EA-induced CPP was completely blocked by naloxone. The time spent in EA-associated compartment was significantly less in the Nx + EA group than that of saline + EA group (*P* <  .01), but not significantly different than that of Blank and Restraint group (*P* >  .05).

### 3.3. EA-Induced CPP Impaired by CB1 Antagonist AM251

**Figure 3:**
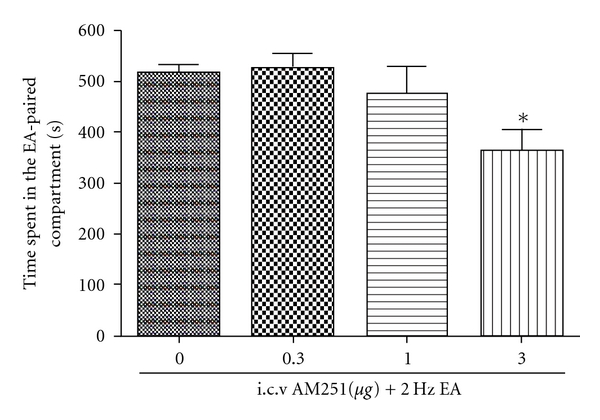
The first column is a control group. The dose “0” means i.c.v. DMSO as a solvent of AM251. EA-induced CPP blocked by i.c.v. injection of AM251 at 3 *μ*g, but not 0.3 and 1.0 *μ*g dose. *P <  .05, compared with DMSO + EA group; (*n* = 9–11). One-way ANOVA followed by Student-Newman-Keul's test.

To explore whether endocannabinoids were involved in EA-induced CPP, we investigated AM251, a CB1 antagonist, on the establishment of EA-induced CPP. Forty-four rats have been divided into four groups. Fifteen minutes before each EA session, the DMSO + EA group were treated with DMSO (4 *μ*l DMSO, i.c.v.), and the other three groups with different doses of AM251 (0.3, 1, 3 *μ*g per 4 *μ*l per rat), respectively. The results were shown in Figure 3. An i.c.v. injection of AM251 (at 3 *μ*g, but not 0.3 and 1.0 *μ*g dose) blocked the establishment of EA-induced CPP.

### 3.4. Effect of Dopamine Receptor Antagonist on EA-Induced CPP

**Figure 4:**
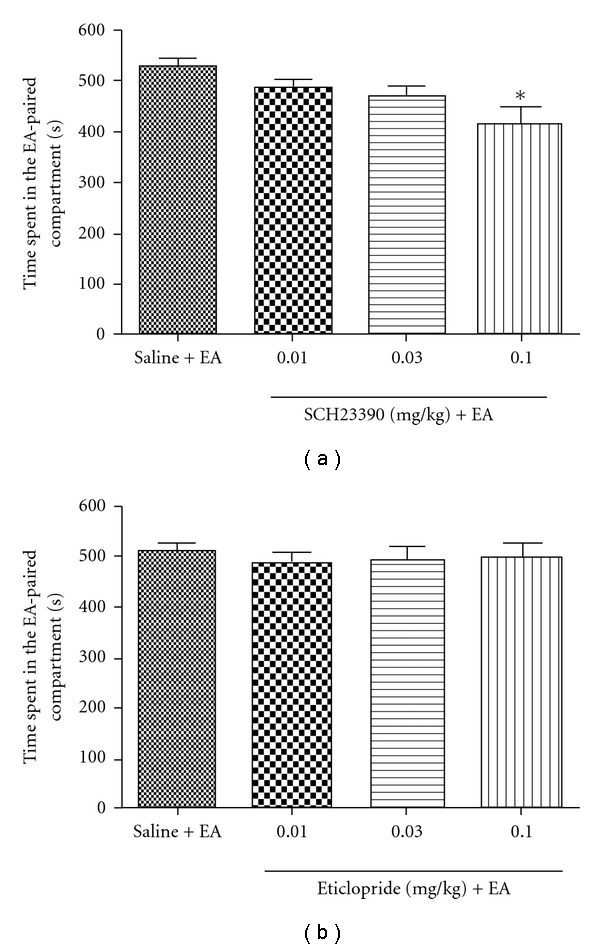
Effects of dopamine receptor antagonist on the expression of CPP induced by EA. EA-induced CPP was blocked by D1 receptor antagonist (SCH23390, 0.1 mg kg^−1^, i.p.), but not by D2 receptor antagonist; (*n* = 9–12). One-way ANOVA followed by Student-Newman-Keul's test.

As shown in Figure 4, D1 dopamine receptor antagonist SCH23390 or its vehicle was injected 15 min before each EA, and it was indicated that SCH23390 dose-dependently reduced the time spent in the EA-paired compartment. In contrast, D2 antagonist eticlopride failed to affect the expression of EA-induced CPP.

## 4. Discussion

Results in the present study indicate that 2 Hz EA *per se* could induce conditioned preference to the EA-associated compartment in male Sprague-Dawley rats, and this CPP could be impaired by pre-conditioning administration of naloxone (against *μ*-opioid receptor), AM251 (CB1 antagonist) and SCH23390 (D1 antagonist). These findings suggest that 2 Hz EA may exert a reward effect via activation of endogenous opioid-, cannabinoid- and dopamine-systems in the brain's reward circuitry.

An accelerated release of enkephalin and *β*-endorphin during low frequency EA stimulation has been shown repeatedly in previous studies [8, 18], and the endogenous opioids triggered by the EA may produce a reward effect, which is supported by a recent study showing that enkepahlins and endorphins, but not dynorphin, are involved in the modulation of conditioned food reinforcement [10]. The sites of action of opioids in rewarding were further identified to the hot spots in nucleus accumbens (NAc) and ventral pallidum (VP). Opioids in NAc and VP work together to promote and reward hedonic impact (“liking”) to incentive motivation (“wanting”), whereas opioids in NAc work cooperatively with other brain areas such as lateral hypothalamus, amygdala and many other structures to execute “wanting” behavior [11]. Also, the results in the present study showed the dose of naloxone (2 mg kg^−1^), sufficient to block the *μ*- and *δ*-opioid receptors [19], could block the EA-induced CPP, but had no significant CPA property, which is inconsistent with results of the previous studies [20, 21]. This discrepancy may mainly be due to the difference at the time point of naloxone administration. In their works, naloxone (1 or 2 mg kg^−1^) was injected 2 min before the rats or mice were putting into the chamber. Whereas in our study, the rats received naloxone injection 15 min before the EA session that lasted for another 30 min, so the rats were put into the CPP training chamber 45 min after the naloxone injection. In fact, Braida et al. [21] have demonstrated that if rats underwent the conditioning 30 min after naloxone (2 mg kg^−1^, i.p.) treatment, they would not express aversive properties anymore.

Endocannabinoid system in the brain has been shown to interact with the opioid system in many physiological activities such as anti-nociception [22], sedation/catalepsy [23–26] and reward [21]. The latter suggests that the rewarding properties of cannabinoids and opioids might be functionally linked. However, it has still been controversial whether they are connected in serial or parallel. There was evidence that the acquisition of CPP induced by morphine (4 mg kg^−1^) could be dose dependently blocked by pre-pairing administration of CB1 antagonist SR141716A (0.03–3 mg kg^−1^) in rats [12, 13], suggesting that the endocannabinoid system may be downstream of opioid. It was also reported that CB1 agonist CP55,940 could elicit CPP at a dose of 20 *μ*g kg^−1^, which in turn was fully antagonized by pre-treatment with 2 mg kg^−1^ naloxone [22], implying that the endocanabinoind system is upstream of endogenous opioid system. No indication can be found from our study in this regard, since EA's reward effect could be blocked by either CB1- or *μ*-receptor antagonist.

Both endogenous opioid peptides and opioid drugs are known to enhance the release of DA in the NAc during the induction of reward. So if EA could activate both opioid system and endocannabioid system, it may also activate DA system. Indeed, it has been reported that EA can modulate the production of dopamine in the CNS [27]. Concerning the D1 antagonist SCH23390, it has been documented that high-dose of SCH23390 (0.5 mg kg^−1^) can induce CPA, while in our study we used the relatively small dose (≤0.1 mg kg^−1^), which has been shown not to induce CPA [28]. The result in the present revealed that the 2 Hz EA-induced CPP can be blocked only by D1 dopamine receptor antagonist SCH23390, but not D2 antagonist Eticlopride, at a physiologically relevant concentration, it shows that the D1 receptor may play a more important role in EA-induced CPP. Since mice lacking D2 receptors could not develop opioid-CPP, yet behave much the same as wild-type mice in CPP paradigm when food is used as rewarding stimulation [29], it seems that EA is more like a natural (e.g., food) reward than a drug reward. Concerning the intensity of the CPP, while EA-induced CPP is a statistically significant and reproducible phenomenon, it is milder than morphine-induced CPP.

Taken together, since the findings suggest that 2 Hz EA could induce CPP, both endogenous opioid system and endocannabinoid system were implicated. Activation of the two systems may modulate dopamine system, which enhances the associative learning in the rewarding procedure.
